# Evaluation of the Immunostimulant Effect of Microvesicles of *Lactobacillus acidophilus* Isolated from Wild Rats

**DOI:** 10.3390/microorganisms13061341

**Published:** 2025-06-10

**Authors:** Pamela I. Pérez-Martínez, Viridiana Gutiérrez-Espinosa, Christian Ávalos-Gómez, Mireya De la Garza-Amaya, Alejandro Vargas-Ruíz, Rosa I. Higuera-Piedrahita, Ernesto Marín-Flamand, Cristal D. Lonngi-Sosa, Francisco R. González-Díaz, Hugo Ramírez-Álvarez, Cynthia González-Ruíz

**Affiliations:** 1 Molecular Pathology Veterinary Laboratory, Multidisciplinary Research Unit, Facultad de Estudios Superiores Cuautitlán, UNAM, Cuautitlán Izcalli CP 54714, State of Mexico, Mexico; izateami@hotmail.com (P.I.P.-M.); viridianagutierrezmvzcl@gmail.com (V.G.-E.); styfler18@hotmail.com (A.V.-R.); rhiguera05@comunidad.unam.mx (R.I.H.-P.); marflamvz@hotmail.com (E.M.-F.); skriztal@gmail.com (C.D.L.-S.); folodro2013@gmail.com (F.R.G.-D.); 2 Virology, Genetics and Molecular Biology Laboratory, Facultad de Estudios Superiores Cuautitlán, UNAM, Cuautitlán Izcalli CP 54714, State of Mexico, Mexico; 3 Centro de Investigación y de Estudios Avanzados del IPN, CINVESTAV Gustavo A. Madero, Ciudad de Mexico CP 07360, CDMX, Mexico; chris8814@hotmail.com (C.Á.-G.); mireya.dela.garza@cinvestav.mx (M.D.l.G.-A.)

**Keywords:** microvesicles, probiotics, *Lactobacillus acidophilus*, immunostimulant, cytokines

## Abstract

Lactic acid bacteria are components of the gastrointestinal tract microbiota in both humans and animals and are widely used as probiotics. *Lactobacillus* is the most closely related genus to probiotic activity. It is capable of releasing membrane microvesicles (MVs), whose primary functions include carrying and transmitting antigens to host tissues and modulating host defense responses. In the present study, MVs were isolated from *Lactobacillus acidophilus* resident in the ileum of free-living rats, and their immunostimulant effect was evaluated in two biological models. MVs were characterized using SDS-PAGE electrophoresis, electron microscopy, and nanoparticle tracking analysis. In the first model, the immunostimulatory effect of MVs was evaluated on ovine abomasal explants, which had been previously stimulated with MVs and then challenged with third-stage larvae of *Haemonchus contortus*. This resulted in a decrease in the percentage of larval association and favored the migration of inflammatory cells to the infection site. In the second model, the macrophage cell line RAW 264.7 was stimulated with MVs to evaluate the expression of transcripts encoding IL-1β and TNF-α. MVs isolated from *L. acidophilus* demonstrate immunostimulatory and probiotic effects in the two biological models assessed. This suggested that the MVs possess similar immunostimulatory effects as those reported for the parent bacteria.

## 1. Introduction

Lactic acid bacteria (LAB) are a broad group of microorganisms commonly used as probiotics. *Lactobacillus acidophilus* is the most well-known strain of the Lactobacillus genus since it has been the most used and studied bacterium over time [1]. LAB is widely found in human and animal mucosae, as well as in fermented foods [2,3]. These microorganisms are associated with immunomodulatory functions and the capacity to reduce the risk of infection and death by pathogens in the gastrointestinal tract (GIT) of mammals [4]. Probiotics are live microorganisms that have beneficial effects on the host when they are administered orally. Scientific evidence indicates that the molecules and cellular structures released by these microorganisms possess significant probiotic properties [5,6,7].

All prokaryotic cells have developed numerous ways to transfer proteins to the environment, which primarily involve the assistance of dedicated protein secretion systems. In that way, the secreted membrane microvesicles (MVs) have become one of the most critical mechanisms of bacterial secretion systems and intercellular communication. MVs are blebs from the outer membrane (OMVs) of Gram-negative bacteria and the cytoplasmic membrane (MVs) of Gram-positive bacteria, which are secreted during all growth phases of both bacteria [8,9].

Compared to the extensive study of OMVs, MVs have been of relatively recent interest, so the mechanisms of biogenesis are still being elucidated [9]. However, it is believed that the formation of MVs may be stimulated by the weakening of the cell envelope through enzymes that hydrolyze peptidoglycan or by β-lactam antibiotics [9,10,11]. In addition to the above, the use of sublethal concentrations of antibiotics that interfere with cell wall synthesis and protein production, such as penicillin [10], ampicillin [11], and gentamycin [12], results in a significant increase in MV production.

Regarding the genus *Lactobacillus*, MVs production has been described in *L. rhamnosus* JB-1 [7], *L. plantarum* [13], *L. acidophilus* [14], *L. reuteri* [15], and *L. casei* BL23 [16]. Immune regulation by MVs from *Lactobacillus* has been proposed to play a role in signaling between probiotic intestinal bacteria and their mammalian hosts [7]. MVs’ immunomodulatory effects are related to their interaction with different immune and non-immune cells through pattern recognition receptors, such as Toll-like receptors (TLR) and NOD-like receptors (NLR), as well as various pathogen-associated molecular patterns (PAMPs) [17]. MVs from different bacterial species have been shown to increase the production of proinflammatory cytokines. For instance, *Staphylococcus aureus* MVs [18] stimulate IL-1β and IL-18 production in murine models, *Escherichia coli* C25 enhances IL-8 production in intestinal epithelial cells [19], and *Akkermansia muciniphila* induces IL-6 secretion [20]. For *Lactobacillus-derived* MVs, *L. casei* has been reported to increase IL-6 in human intestinal epithelial cells [21], and *L. reuteri* DSM-17938 promotes motility in mouse intestines [15]. The important immune response that MVs stimulate in the host has facilitated the development of acellular vaccines against pathogenic bacteria, such as *Neisseria meningitidis* [22], *Vibrio cholerae* [23], *Burkholderia pseudomallei* [24], as well as *Mannheimia haemolytica* A1 [25] and A2 [26].

The use of probiotics in animal production and health is becoming increasingly relevant [27,28], because the supplementation of probiotics in the diet can improve animal health and performance, through contributions to gut health and nutrient absorption [29,30], as well as to reduce the use of antimicrobials to which microorganisms have become increasingly resistant. The primary objective of probiotics is to modulate the host immune system, thereby preventing and treating infections [31]. In the livestock industry, it has been reported that the administration of probiotics enhances milk production in Holstein Friesian cows [32,33], while in other species, such as pigs, it promotes microbiota diversity and weight gain [34,35,36]. In poultry, studies have shown that probiotics can improve broiler and egg production [37,38]. Probiotics have not been limited to animal production. Still, they are also used in pets, such as dogs and cats, which promotes the diversity of microbiota, improves the immune response, and helps control diarrhea [29,39,40].

In a previous study, the production and characterization of MVs from *Lactobacillus* genus bacteria isolated from the gastrointestinal tract (GIT) of free-living rats were studied [41]. The relevance of working with *Lactobacillus* isolated from this rodent is justified because it is exposed and resistant to many pathogens that infect humans and food animals without developing disease, which can be attributed to its intestinal microbiota [42,43,44]. Lonngi et al., 2022 previously reported a bactericidal effect of 30 µg of MVs from *L. acidophilus* isolated from the ileum of free-living rats on the growth of *Salmonella enterica* serovar Typhimurium (ATCC 154) and a field strain of *E. coli* [41].

On the other hand, parasitic diseases affecting the livestock industry are a persistent and challenging problem due to the resistance that parasites have developed to the various drugs intended for their control. One of the most significant ovine gastrointestinal tract (GIT) diseases is caused by *Haemonchus contortus*. This hematophagous parasite induces anemia and mortality in animals, even when it triggers a strong Th2 immune response [45,46,47]. The life cycle of this parasite is direct, and animals become infected when they consume feed contaminated with third-stage larvae (L3), the infective stage. Once in the abomasum, it develops into L4 and L5, remaining in this stage until it reaches adulthood [48]. However, there are no reports of the effects of MVs of probiotic bacteria on L3 of *H. contortus*.

Although the microbicidal and immunostimulant effects of probiotics are well established, there are few studies on the impact of MVs from *L. acidophilus*. Nevertheless, no information is available for the minimum viable concentrations of *L. acidophilus* MVs isolated from free-living rats. Bacterial MVs, in general, have demonstrated their potent ability to carry biologically active antigens, which efficiently stimulate the immune system. This study aimed to determine the immunostimulatory capacity of MVs from *L. acidophilus* on two *in vitro* biological models: the first, using ovine abomasal tissue explants, and the second, macrophage cell line cultures (RAW 264.7), to assess its potential as a probiotic strategy against *H. contortus*. The present study is the first report on the effects of *L. acidophilus* MVs isolated from free-living rats against pathogens that affect animal production and cellular responses.

## 2. Materials and Methods

### 2.1. Cells and Strains

In a previous work, *Lactobacillus acidophilus* ATCC^®^ 314 (KWIK-STIK, Microbiologics, Charlottesville, VI, USA) and *L. acidophilus*, a field isolate from the free-living rats’ ileum, captured in the Metropolitan area in Mexico City, Mexico, and characterized using real-time PCR and the API 50CHL system (Biomérieux, Lyon, France) [41], were used.

The macrophage cell line RAW 264.7, gamma NO (-) (ATCC, Charlottesville, VI, USA) was used, and *Haemonchus contortus* strain third-stage larvae (L_3_) were obtained from two donor sheep, previously infected with 5000 L_3_ of *H. contortus* FESC UNAM strain. Recovery of eggs from *H. contortus* was performed using the methodology described by Reséndiz et al., 2022 [49].

### 2.2. Animals

Ovine Columbia breed free-grazing male adults were used to obtain larvae (23.5 ± 2 kg of body weight) (FES Cuautitlán, Cuautitlán Izcalli, State of Mexico, Mexico). Ovines were maintained indoors in metabolic cages and supplied with hay, commercial concentrate, and water ad libitum. The animals were housed in accordance with the care and welfare guidelines of the Mexican Official Rule NOM-051-ZOO-1995 [50]. The ovine abomasal tissue was donated from animals slaughtered in a slaughterhouse.

### 2.3. Obtaining and Characterization of MVs

*Lactobacillus acidophilus* strains, both field and ATCC, were grown in 250 mL of Man, Rogosa, Sharpe (MRS) broth (Becton Dickinson, Mexico)/37 °C/24 h in the absence of CO_2_. In the logarithmic growth phase (3.5 h), 10 µg/mL gentamicin was added to induce the release of MVs, and the mixture was incubated overnight. The culture was centrifuged at 2500× *g* for 15 min at 4 °C to remove biomass (whole bacteria, WB) and recover the supernatant, which was then filtered through 0.45 µm and 0.22 µm diameter Millipore membranes to remove the Whole bacteria (WB). The filtered supernatant (cell-free supernatant, CFS) was ultracentrifuged at 150,000× *g* for three hours at 4 °C. The resulting pellet corresponded to MVs, which were resuspended in 1 mL of 10 mM HEPES and frozen at −70 °C until use.

### 2.4. Characterization of MVs of Lactobacillus acidophilus

#### 2.4.1. Nanoparticle Tracking Analysis

Nanoparticle tracking analysis (NTA) was performed using a Nanosight NS 300 (Malvern Panalytical, Malvern, UK). The 1 mL sample of MVs was diluted 1:100 in 1X phosphate-buffered saline (PBS) (filtered through a 0.22 μm filter). From this sample, an NTA was performed, resulting in the recording of three 30 s videos with an sCMOS camera to determine the size and particle concentration at the National School of Biological Sciences, Santo Tomás Unit, National Polytechnic Institute (IPN).

#### 2.4.2. Transmission Electron Microscopy of MVs

For Transmission Electron Microscopy (TEM), the MVs were resuspended in cacodylate buffer, and 10 to 15 μL was placed on nickel grids that had been previously treated with Formvar and coated with carbon, allowing them to set for 15 min (Electron Microscopy Sciences, USA). Afterwards, the excess sample was removed with filter paper, and the grid was carefully dried. Then, 1% phosphotungstic acid (PTA/ Electron Microscopy Sciences, Sigma-Aldrich, Burlington, MA, USA) was added at pH 6.0 for 90 s. Finally, samples were observed under a transmission electron microscope (JEM-1400, JEOL, Peabody, MA, USA) at the Research and Advanced Studies Center of the National Polytechnic Institute (CINVESTAV), located at the Zacatenco Unit.

### 2.5. SDS-PAGE

Protein samples were quantified with the Bradford method (Bio-Rad, Hercules, CA, USA) with linear regression and bovine serum albumin (BSA) standard curve. Protein samples (2 µg) of MVs, WB, and CFS from both strains were analyzed by denaturing polyacrylamide gel electrophoresis using a 12% pre-cast acrylamide gel with the Mini-Protean R Tetra Cell 4 Gel System Kit (Bio-Rad, Hercules, CA, USA), following the manufacturer’s instructions. Samples were denatured by boiling in the presence of a reducing agent (2-mercaptoethanol, Sigma-Aldrich, Massachusetts, USA) before gel loading. The dual-color molecular weight marker 1610374TGX (Bio-Rad, California, USA) was used and run at 100 volts for 2.5 h. Subsequently, the gel was stained using a Silver Stain Pierce kit (Thermo Scientific, Walthman, MA, USA) following the manufacturer’s instructions. The electrophoretic run was evaluated using KODAK MI Software Gel-Logic 100 Imaging System (GL 100), System 1.

### 2.6. Obtaining the L3 Larva Stage from Haemonchus contortus

Fecal samples were obtained directly from the rectum of a male ovine donor. They were cultured in Petri dishes, following the Corticelli–Lai technique for 7 days [51]. Infective larvae were extracted from the fecal material using the Baermann funnel technique [52]. The larvae were cleaned using distilled water, centrifuged at 5000× *g*, and then exsheathed with 0.187% sodium hypochlorite [52]. Finally, L3 was used for subsequent assays.

### 2.7. Culture of Ovine Abomasal Explants

The abomasal tissue was obtained from an ovine slaughtered by the care and welfare guidelines of the Mexican Official Rule NOM-033-SAG/ZOO-2014 [50] and was carefully washed with warm Hank’s medium (Corning, NY, USA). Circular sections of approximately 3 cm^2^ were subsequently cut and placed in six-well culture plates containing 5 mL of warm HANKs, then 5 mL syringes with the needle end removed were placed in the center of each tissue to provide a 13 mm diameter of an isolated zone containing *H. contortus* larvae, according to the technique described by Jackson et al. in 2004 [53]. MVs (30 μg) in 500 μL of 1X PBS were added to the mucosal surface within the isolation cylinder for the field and ATCC strains and incubated at 37 °C for one hour. Subsequently, 0.5 mL of 1X PBS/1500 *H. contortus* larvae were introduced into the cylinder, and pressure was applied to the lid of the six-well plate to ensure an effective seal. Tissues with and without larvae were used as positive and negative association controls, respectively. Cultures were incubated for an additional two hours at 37 °C and 5% CO_2_.

### 2.8. Recovery of L3 Larvae of H. contortus

After incubation, the syringe cylinders and tissues were washed with a 1X PBS solution in 50 mL conical tubes (wash tube) to remove all larvae not associated with the mucosa. The tissue was placed in a second tube (digestion tube) and digested in 50 mL of a 1% pepsin solution in 1% HCl at 25 °C for 72 h. The number of larvae present in 2% aliquots of each sample was counted using a gridded Petri dish under a stereoscopic microscope (HINOTEK, NSZ-405, Ningbo, China) at 100× [53]. The percentage of association in the tissue (digestion tube) was calculated.

### 2.9. Histopathology of the Ovine Abomasal Explants

After the experiment was completed, samples were taken from the region delimited by the syringe and fixed in 10% buffered formalin for 24 h. Subsequently, they were subjected to the paraffin technique and stained with hematoxylin and eosin (H&E) for review under a light microscope [26].

### 2.10. Larval Mortality Assay

A larval mortality assay was performed using 96-well microtiter plates, with 30 μg of MVs from both *L. acidophilus* strains, distilled water serving as the negative control, and 0.5% ivermectin as the positive control. An aqueous suspension (50 µL) containing 100 ± 15 infective larvae was added to each well. Subsequently, 50 µL of MVs and controls were added to each well individually. The plates were incubated at 25 °C in a humidified chamber for 24 h. The total number of larvae (live or dead) in each well was counted using a microscope [49]. Mortality percentages were estimated using the criteria outlined by Olmedo et al. [54].

### 2.11. RAW 264.7 Cell Cultures

Cells of the RAW 264.7 line (1 × 10^6^) were cultured in 24-well culture plates with RPMI 1640 medium high in glucose (ATCC) supplemented with 10% fetal bovine serum (GIBCO, New York, NY, USA), 2 mM L-glutamine (GIBCO, New York, USA), and 1% antibiotics (CAISSON, Rexburg, ID, USA). For the negative control, cells were maintained without treatment, and as a positive control, 2 μg of LPS from *E. coli* O111: B4 (Sigma Aldrich, Massachusetts, USA) was added. MVs (10 μg) of both *L. acidophilus* strains were used to stimulate the cells, which were then incubated for one hour at 37 °C in a 5% CO_2_ atmosphere. Following the incubation period, the cells were detached by raking and suspended in 1 mL of Trizol LS Reagent (Thermo Scientific, Massachusetts, USA) to extract RNA, according to the manufacturer’s instructions.

### 2.12. qPCR

cDNA was obtained using a FastGene Scriptase Basic cDNA Synthesis Kit, NIPPON kit (Nippon Genetics, Tokyo, Japan) according to the manufacturer’s instructions. qPCR for IL-1β and TNF-α was performed using an Agilent Technologies, Stratagene Mx3005P Thermal Cycler, Thermo Scientific, Massachusetts, USA). Primers were designed using Primer3web (v.4.1.0) software [55]; the primer sequences are listed in Table 1. The amplification conditions are briefly described: 1 denaturation cycle of 2 min at 95 °C, followed by 10 s 95 °C, 10 s 60 °C, and 20 s 72 °C for 40 cycles, and melting curves were for 5 s 95 °C, 10 s 60 °C, and 10 s 95 °C. Quantification was performed by relative expression using the hypoxanthine-guanine phosphoribosyltransferase enzyme (HPRT1) as the reference gene, with an annealing temperature of 50 °C.

### 2.13. Statistical Analysis

Data were analyzed based on the repetitions and data distribution; a one-way analysis of variance (ANOVA) and Tukey’s multiple comparison test were used. All statistical analyses were performed using GraphPad Prism 8 software (GraphPad Software, Inc., San Diego, CA, USA). Statistical significance was established at *p* < 0.05.

## 3. Results

### 3.1. Characterization of MVs by Nanoparticle Tracking Analysis and Transmission Electron Microscopy

**Figure 1:**
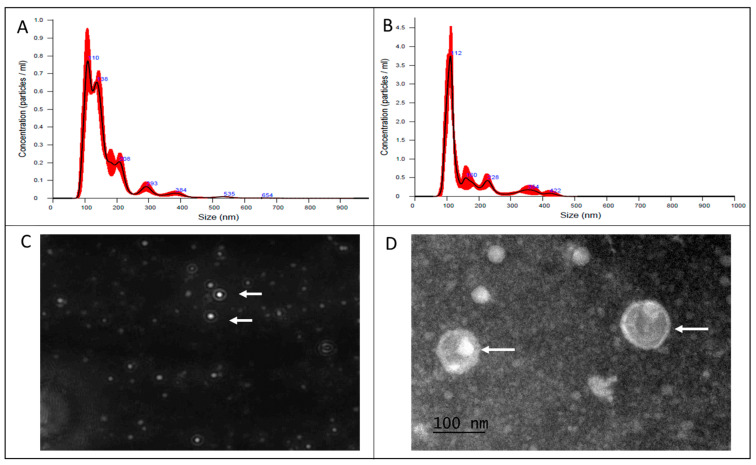
Characterization of extracellular microvesicles (MVs) secreted by *Lactobacillus acidophilus*. Concentration and size distribution [nm] of MVs produced by field (**A**) and ATCC (**B**) *L. acidophilus*, where the red hihglights correspond to the dispersion of the data meanwhile the blue numbers are the size of the nanoparticles. (**C**) Representative frame of one of the videos of *L. acidophilus* in NanoSight, where MVs are observed (white arrow). (**D**) TEM micrograph of MVs produced by field *L. acidophilus* stained with phosphotungstic acid (white arrow).

MVs from the field isolate of *L. acidophilus* have a size of 165.6 ± 9.2 nm in diameter at a concentration of 6.27 × 10^8^ particles/mL, and MVs from the ATCC strain of *L. acidophilus* presented a size of 160.7 ± 11.0 nm at a concentration of 1.65 × 10^10^ particles/mL. Figure 1A,B show the representative histograms obtained by nanoparticle tracking analysis (NTA) for both strains. From one of the field strain videos, a frame was taken where homogeneity in form and size was observed (Figure 1C). However, there is no significant difference in the size and form of the structures between the two strains; this can be attributed to the coalescence capacity of the MVs during the NTA procedure. In the TEM image (Figure 1D), we observed MVs of the field strain, which had an individual diameter of approximately 100 nm. A double-membrane structure was observed, forming the vesicular structure of the MVs.

### 3.2. L. acidophilus MVs Carry Multiple Proteins

**Figure 2:**
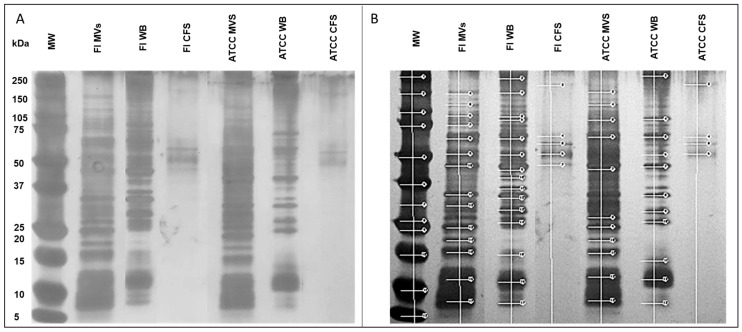
Protein composition of different cell fractions of *Lactobacillus acidophilus* (**A**) SDS-PAGE gel (12%), stained with silver, of proteins from both field (FI) and ATCC strains of *L. acidophilus* from MVs (FI MVS and ATCC MVS), whole bacteria (FI WB and ATCC WB), and cell-free supernatant (FI CFS and ATCC CFS). In all lanes, 25 μg of protein was placed. (**B**) SDS-PAGE 12% gel analysis, with KODAK Gel-Logic 100 system software, where proteins detected by the software are marked with white lines.

From the analysis of SDS-PAGE of proteins found within the MVs, whole bacteria (WB), and cell-free supernatants (CFS) using a 12% polyacrylamide gel (Figure 2A), a distinct pattern is observed between the field-isolated and ATCC strains. Furthermore, it has been observed that there are multiple similar molecular weight proteins (approximately 25 to 75 kDa) that match the whole bacteria and the MVs from both strains. It is essential to note that the lowest molecular weight proteins are not included in the CFS. The molecular weights of the proteins obtained from the different runs were possibly identified using the KODAK MI Software Gel-Logic 100 Imaging System (GL 100), System 1 (Figure 2B). In comparison to previous studies [8], proteins associated with probiotic functions (p40 and 045), as well as cell division (FtsZ, DivlB), ABC transporters (ABC transporter ATP-binding and membrane-spanning protein, as well as Glutamine ABC transporter permease protein), and possible bacteriocins (bacteriocin like proteins) were potentially identified [8]. Some of these proteins were observed in MVs of both strains, the whole bacteria, and CFS, and are represented in Table 2.

### 3.3. Ovine Abomasal Tissue Explants Stimulated with L. acidophilus MVs Decrease Larvae Association

**Figure 3:**
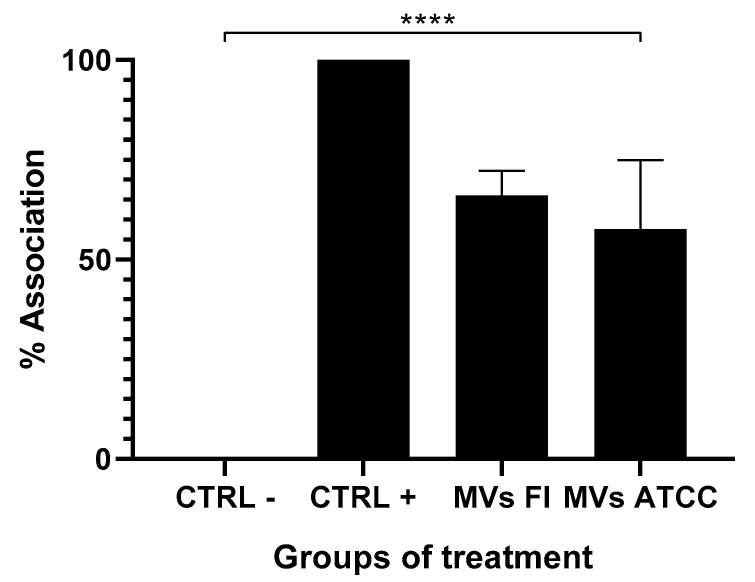
Percentage of *Haemonchus contortus* L3 association in ovine abomasal tissue. Association of *H. contortus* L3 larvae in abomasal tissue of ovine, where abomasal mucosa only (CTRL-), without stimulation of MVs (CTRL+), and stimulated with 30 μg of MVs of *L. acidophilus* field strain (FI) and ATCC strain, a significant decrease in the percentage of association is appreciated. One-way ANOVA *p* = 0.001 (****).

Experiments were performed by stimulating ovine abomasal tissue explants with 30 μg of MVs from the field and ATCC *L. acidophilus*, which were challenged with 1500 L3 larvae of *H. contortus*. After counting the larvae and calculating the percentage of association, a significant decrease (*p* < 0.0001 and *p* < 0.0085) in the percentage of association (Figure 3) in the groups stimulated with MVs of both strains is shown, which was lower than that of the positive control (without MVs) when a one-way ANOVA statistical test was applied.

### 3.4. Stimulation with the Field L. acidophilus MVs Increases the Presence of Inflammatory Cells in the Abomasal Tissue

**Figure 4:**
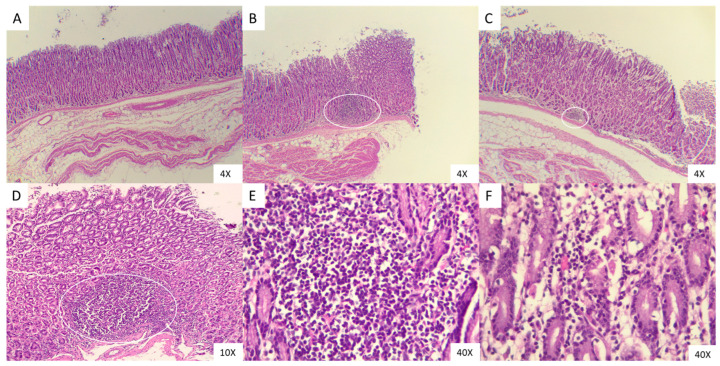
Sections of ovine abomasal tissue stimulated with MV of *Lactobacillus acidophilus* stained with H&E. Ovine abomasal tissue. (**A**) Unstimulated tissue (Ctrl−). (**B**) Tissue challenged with 1500 L3 of *H. contortus* (Ctrl+). (**C**) Tissue stimulated with MVS of *L. acidophilus* field strain. (**D**) Approach 10× to the tissue stimulated with MVS of *L. acidophilus* field strain. Mucosal-associated lymphoid tissue in (**B**–**D**) panels is marked with an oval. (**E**) With a 40× objective, the presence of lymphocytic cells and some macrophages in the submucosal area of the tissue challenged with L3 of *H. contortus*, and (**F**) infiltrate between the crypts of the tissue stimulated with *L. acidophilus* MVs from the field strain.

Histological sections were prepared and stained with H&E to evaluate the explants in contact with MVs and larvae. Ovine abomasal tissue was observed, presenting mainly with foci of lymphocytic infiltrate in the submucosa, and to a lesser extent, macrophages (Figure 4). Histopathological examination of the abomasal mucosa without treatment reveals normal autolysis of the villi, consistent with the post-mortem period (Figure 4A). The treated abamosal with the L3 larvae revealed reactive germinal centers at the submucosal level (Figure 4B) as same as with the MVs from *L. acidophilus* field isolated strain (Figure 4C), with the difference that under 10× and 40× (Figure 4D,E, respectively) objectives, is demonstrating the presence of inflammatory cells infiltrating the interstitium of the crypts and in the lamina propia of the villi (Figure 4F).

### 3.5. L. acidophilus MVs Do Not Kill the L3 Larvae of H. contortus

**Figure 5:**
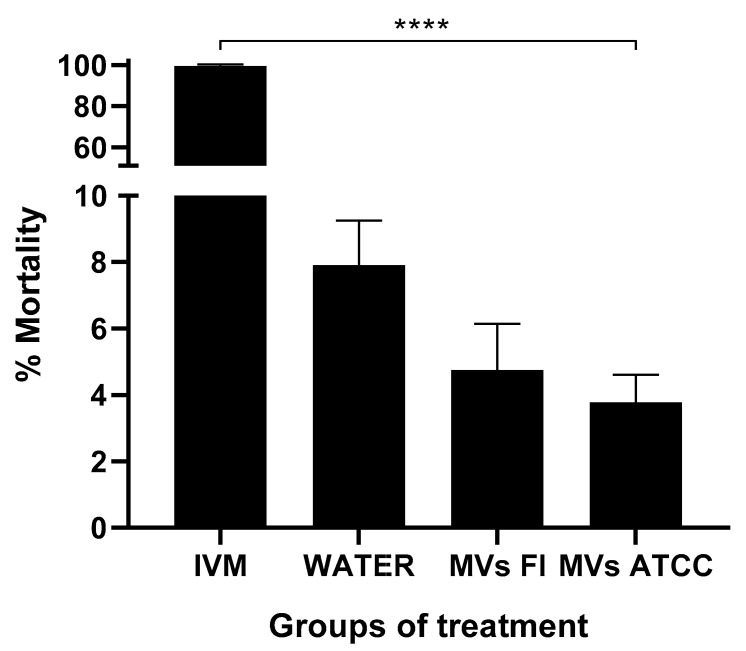
Percentage mortality of *Haemonchus contortus* L3. Larvae mortality assay of *H. contortus* L3 incubated with 30 μg of MVs of *L. acidophilus* field strain (FI) and ATCC strain. As a positive control, 0.05% ivermectin (IVM) was used. It showed a 98% mortality rate among the larvae, resulting in a significant difference compared to the negative control (water) and the treatments with both *L. acidophilus* MV strains. One-way ANOVA *p* = 0.001 (****).

To determine whether the decrease in the percentage of association observed was due to the effect of *L. acidophilus* MVs on L_3_, a larval mortality assay was performed. In the positive control group treated with 0.05% ivermectin, the highest percentage of L3 death (98%) was observed, while the negative control group treated with water showed less than 10% (Figure 5). The mortality percentages of the groups treated with MVs from both strains were similar (3.78% for ATCC and 4.75% for the field strain) to those of the negative control group, although a significant difference was observed (*p* < 0.0001; one-way ANOVA) compared to the group treated with ivermectin.

### 3.6. L. acidophilus MVs Activate Macrophages (RAW 264.7)

**Figure 6:**
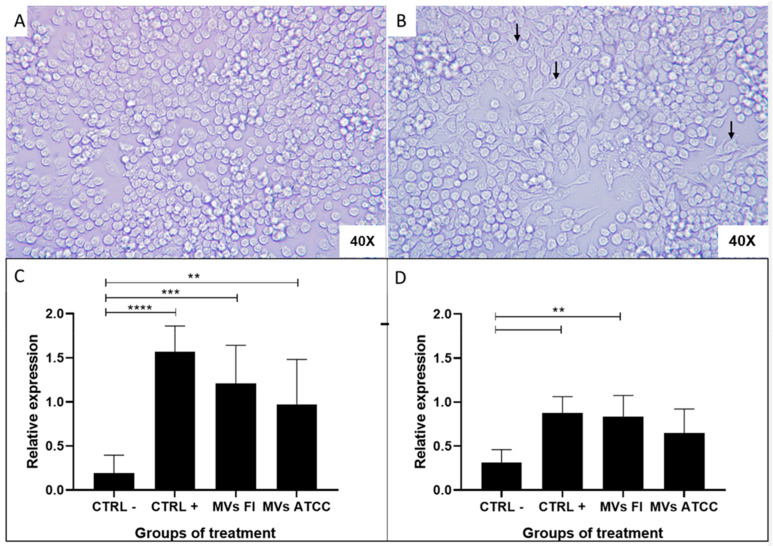
RAW 264.7 cells stimulated with *Lactobacillus acidophilus* MVs and relative expression of IL-1β and TNF-α mRNA. RAW 264.7 cells (**A**) untreated cells (negative control)/40×, (**B**) stimulated cells with 10 μg of MVs obtained from a field strain (MVs FI)/40×. In activated macrophages, more pseudopods are observed (black arrow), (**C**) Relative mRNA expression of TNF-α and (**D**) Relative mRNA expression of IL-1β. Stimulation with MVs increases the relative messenger expression of both cytokines compared to the negative control. Tukey’s multiple comparisons test *p* 0.0084 (**), *p* 0.0008 (***), *p* < 0.0001 (****). Messenger RNA (mRNA), Interleukin 1 beta (IL-1β), Tumor necrosis factor alpha (TNF-α).

When macrophages of the RAW 264.7 cell line (Figure 6A) were stimulated with MVs from both L. acidophilus strains, the cells modified their morphology, exhibiting a greater number of pseudopodia (Figure 6B), which suggests macrophage activation. After incubating the cells for one hour, RNA was extracted, and quantitative PCR (qPCR) was performed. An increase in the expression of IL-1β (Figure 6C) and TNF-α (Figure 6D) was found, compared with the positive control group, which received LPS. In addition, relatively higher messenger expression was observed upon stimulation with *L. acidophilus* MVs from the field strain compared to those from the ATCC strain.

## 4. Discussion

Rats are important carriers of pathogenic bacteria that affect both humans and animals, such as *Salmonella*, *Escherichia coli*, *Campylobacter*, and *Listeria*, among others [56]. However, in most cases, they do not exhibit signs or symptoms of infection, partly due to their microbiota [56,57]. Studies focused on the microbiota of both free-living and laboratory rats describe *Lactobacillus* as one of the most abundant genera along the gastrointestinal tract [58,59]. Due to the importance of this genus, it was used in the present study.

Numerous studies have reported beneficial effects of *L. acidophilus* [1,4,5,8,27,28,36]. In the present study, we have demonstrated that MVs from this bacterium could stimulate an immune response in the analyzed biological models.

The production of *L. acidophilus* MVs was confirmed by TEM and NTA, with diameters of approximately 160 nm, similar to those previously reported by Dean et al. [14] for *L. acidophilus* ATCC 53544, which ranged from 100 to 150 nm. Studies analyzing proteins carried in MVs of different bacterial genera, both Gram-positive and Gram-negative, have demonstrated the extraordinary capacity of these structures to conform to proteins from the wall or the external membrane of origin, as well as proteins from the cytoplasm or in transit between the periplasm and lumen of MVs during formation [6,60,61]. The proteins carried in the MVs of both *L. acidophilus* strains were analyzed by SDS-PAGE, which demonstrated that a significant number of protein bands of different molecular weights were shared between them. Dean et al. [14] characterized *L. acidophilus* ATCC 53,544 MVs and identified more than 80 protein components, including bacteriocins and ABC transporters. In the present study, low-molecular-weight proteins ranging from 10 kDa to 6 kDa were identified, like those reported by Dean as possible bacteriocins, which were named bacteriocin-like substances (BLS) [14,62]. In addition, proteins of 32 kDa and 64 kDa, which, due to their molecular weight, coincided with the cell division protein (DivIB) and FtsZ protein [homologous to eukaryotic tubulin], respectively, are known to interfere with cell division [14,63]. The functions of the 40 kDa and 75 kDa proteins in the complete bacterium are reported to be p40 and p75, respectively, which are associated with cell communication, epithelial adhesion, anti-apoptotic effects, and immunomodulatory capacity [64,65,66]. Likewise, proteins of 54 kDa and 59 kDa were identified, which could correspond to ABC transporters, ATP-binding membrane-spanning proteins, and Glutamine ABC transporter permease proteins, respectively. All of these proteins are important for bacterial metabolism [14,62]. However, none of the proteins reported in this study were sequenced and identified.

In explants of ovine abomasal tissue, the association of L3 of *H. contortus* with MVs was evaluated. Over the 3 h duration of the experiment, penetration of the larvae into the mucosa was not observed; therefore, for the purposes of this study, we will only refer to an association phenomenon. *L. acidophilus* MVs from a field isolate and an ATCC reference strain inhibited the association of L_3_ larvae of *H. contortus* with the ovine abomasal explant tissue. Huang, 2021, Martín, 2020, and Gao, 2009 reported that bacteriocins purified from both Gram-positive and Gram-negative bacteria have been shown to affect other pathogenic bacterial genera and inhibit viruses and parasites [60,67,68]. For example, AS-48 is a peptide synthesized by *Enterococcus faecalis* that has a bactericidal effect on many bacteria and reduces the number of *Trypanosoma cruzi* by causing depolarization of the mitochondrial membrane and favoring the production of reactive oxygen species in the parasite [60,67,68]. Notably, no reports have been recorded on the effects of the bacteriocins present in *L. acidophilus* MVs on any parasite.

To test the effect of *Lactobacillus* MVs on the viability of L3 of *H. contortus*, a larval mortality assay was performed. The MVs did not affect the larva viability, as the percentage of mortality was the same as that of the negative control, all contrary to what was observed with ivermectin, which showed a mortality rate of 98% in the larvae since it is a potent antiparasitic effective against *H. contortus* [69]. This suggests that the decrease in larval association with abomasal tissue may be due to MVs’ stimulation of the abomasal mucosa rather than their direct effect on the larvae. The metabolic derivatives of *Lactobacillus* contain various biologically active compounds, including antimicrobial peptides and immune system signaling molecules (PAMPs) [70,71,72], to which the observed effects can be attributed. In 2007, Terefe et al. reported that the abomasal mucosa can synthesize proinflammatory cytokines, including IL-4, IL-5, and IL-13, in response to *H. contortus* infection [73]. IL-13 favors mucosal contractility and mucus production in the tissue, reducing the mobility of L3 larvae, which contributes to the non-association of the parasite [74].

Ovine abomasal tissue analyzed by histopathology showed the presence of mononuclear cells in the interstitium of the crypts and base of the villi in explants previously stimulated with *L. acidophilus* MVs. Simpson et al. [75] demonstrated, through histopathological evaluation of ovine abomasal tissue sections from animals infected with L3 of *H. contortus*, the presence of foci of inflammatory infiltrates confined to the submucosa. Ex vivo studies have described the presence of immune cells in the submucosa of the abomasum without the infiltrate moving to the mucosa. In contrast, the capacity of the MVs in this study to stimulate immune cells favored their migration, which is like the report by Li, 2005, where the supernatants of *L. acidophilus* cultures contained only cellular structures and metabolic derivatives that favored chemotaxis and proliferation of immune cells as well as angiogenesis in chicken embryonic tissues in vitro [76].

Ovine are generally prone to gastrointestinal parasitism because they feed on pastures contaminated with L3 stage larvae of *H. contortus*. In Mexico, its prevalence ranges from 17.5% to 57% among free-grazing animals [77,78,79]. In the animals used in the present study, adult nematodes of *H. contortus* were found in the abomasum of grazing animals, which explains the abundant inflammatory infiltrates found in the histological sections [80]. Constant exposure to *H. contortus* in sheep enhances the development of resistance to subsequent infections by this parasite and others. This is because the host immune system rapidly rejects the association of L3 by a phenomenon known as immune exclusion, wherein immune cells such as T lymphocytes and mast cells release protein compounds such as galectin and histamine that prevent the establishment of L3 in the crypts of the mucosa [80,81,82] by immunomodulation mechanisms. This phenomenon could explain the observed percentage of L3 association described in the present study, as well as that reported in other in vivo [83], in vitro [53], and ex vivo [81] studies.

The stimulation of the host immune system is a primary function of probiotics and their derivatives. In the present study, it was demonstrated that *L. acidophilus* MVs from the field strain can stimulate the murine macrophage RAW 264.7 cells. Morphological changes were observed in cells stimulated with both LPS (control+) and MVs, indicating cell activation, such as the extension of pseudopodia, a characteristic feature of this cell line [84]. The expression of transcripts encoding proinflammatory cytokines, such as IL-1β and TNF-α, was identified. Macrophages primarily secrete IL-1β and TNF-α, and these cytokines have a synergistic effect on inflammation in both infectious and non-infectious processes. Their primary effects include chemotaxis of immune cells, cell activation, and stimulation of cytokine production, such as IL-6, IL-8, and MCP-1 [85].

The presence of complete bacteria and some antigens secreted by them activates macrophages to synthesize proinflammatory cytokines [86,87,88]. One of the most studied components is peptidoglycan, the main component of the cell walls of Gram-positive bacteria. As MVs are formed from the elongation of the membrane, they carry structural elements, including those of the cell wall [14], which act as PAMPs responsible for stimulating the cells in vitro. The whole cell of *L. acidophilus* has been reported to favor the expression of IL-1β in macrophages *in vitro* [89,90,91]. However, Li et al. found that the CFS of *L. acidophilus* cells could increase TNF-α expression in chick embryonic tissues [71]. Studies have been performed with the MVs of other strains of *Lactobacillus*, as is the case with Vargoorani et al., who used MVs derived from *L. casei* and observed an increase in the production of IL-6, not IL-1β, in cultures of human intestinal epithelial cells [21]. In mice orally administered with *L. plantarum*, TNF-α, and IL-6 levels decreased [92]. These studies demonstrate that probiotic bacteria and their derivatives can modulate the immune system and the inflammatory response by increasing or decreasing the production of cytokines. Although they all belong to the same genus, not all species act in the same manner.

The results obtained in this study demonstrate that the MVs of *L. acidophilus* possess a probiotic capacity, stimulating the host immune system, as reported with the use of whole bacteria. Despite this, there is no significant statistical difference between the field-isolated and ATCC strains in the percentage of association of the *H. contortus* larvae; however, there is a tendency for a decrease in the rate with the field-isolated strain. On the other hand, regarding cytokine expression, there is no statistical difference between the strains; however, a significant difference is observed between the negative control and the field-isolated strain. This could be due to the origin of the strain (wild rat) [56,57], but furthermore, studies are necessary to corroborate this. These results enabled us to consider the potential use of these compounds in preventing and controlling infections. MVs have the advantage of not having the capacity to replicate; therefore, they cannot cause disease and can be administered to immunocompromised patients or patients treated with antibiotics, contrary to what has been reported with the use of probiotic bacteria, where sepsis occurs even after their administration [70,92,93].

## 5. Conclusions

*Lactobacillus acidophilus* MVs from the field and the ATCC were shown to have probiotic and immunostimulatory effects on ovine abomasal tissue, decreasing the association with L3 larvae of *H. contortus*. While in cell culture of a murine macrophage line (RAW 274.6), MVs activated the cells by increasing the expression of proinflammatory cytokine transcripts IL-1β and TNF-α. No statistical difference was found between the effects of the two strains; however, a more pronounced trend in the effect was observed with the field strain.

## Figures and Tables

**Table 1:** Primer sequence for the RAW264.7 cell genes.

Primer Gene	Sequence [5′-3′]	Alignment Temperature	Length [bp]
IL-1β	Fw: GTGTGTGACGTTCCCATTA Rv: CGTTGCTTGGTTCTCCTTGT	62 °C	170
TNF-α	Fw: TATGGCTCAGGGTCCAACTC Rv: CTCCCTTTGCAGAACTCAGG	62 °C	174
HPRT1	Fw: ATTCCCAACAGACAGACAGACAGAA Rv: TTAGGTCGGAAGGCATCAT	50 °C	224

Fw—forward primer; Rv—reverse primer; IL 1β—interleukin one beta; TNFα—tumor necrosis factor alpha; HPRT1—hypoxanthine-guanine phosphoribosyltransferase enzyme; bp—base pairs.

**Table 2:** Proteins identified for SDS-PAGE 12% for MVs, whole bacteria, and cell-free supernatants of the field strain and ATCC of *Lactobacillus acidophilus*.

Proteins		Fractions					
Description	PM/ kDa	FI MVs	FI WB	FI CFS	ATCC MVS	ATCC WB	ATCC CFS
p75	75	✓	✓	-	✓	✓	-
FtsZ protein	64	✓	✓	✓	✓	✓	✓
ABC transporter: ATP-binding and membrane-spanning protein	59	✓	-	-	-	-	-
Glutamine ABC transporter permease protein glnP	54	✓	✓	✓	✓	✓	✓
p40	40	✓	-	-	✓	-	-
Cell division protein DivIB	32	✓	-	-	✓	-	-
Bacteriocin-like proteins BLP	10	✓	✓	-	✓	✓	-
	9	✓	✓	-	✓	✓	-
	6	✓	-	-	✓	-	-

The table shows the proteins of both *L. acidophilus* strains, field strain [FI] and ATCC, present [✓] or absent [-] obtained from the fractions: MVs [FI MVS and ATCC MVS], whole bacteria [FI WB and ATCC WB], and cell-free supernatant [FI CFS and ATCC CFS]. By analyzing the molecular weights reported by the KODAK Gel-Logic 100 system software, the proteins were compared with those reported in the literature.

## Data Availability

The data from the results obtained in this investigation are available on the ZENODO repository at https://doi.org/10.5281/zenodo.10999677.

## Abbreviations

The following abbreviations are used in this manuscript:

MVs	Microvesicles
GIT	Gastrointestinal tract
LAB	Lactic acid bacteria
L3	Stage 3 larva
WB	Whole bacteria
CFS	Cell-free supernatant
BLS	Bacteriocin-like substances
FI	Field Isolated
